# Minimally Invasive Treatment of Infrabony Periodontal Defects Using Dual-Wavelength Laser Therapy

**DOI:** 10.1155/2016/7175919

**Published:** 2016-06-02

**Authors:** Rana Al-Falaki, Francis J. Hughes, Reena Wadia

**Affiliations:** ^1^Al-FaPerio Clinic, 48A Queens Road, Buckhurst Hill, Essex IG9 5BY, UK; ^2^Department of Periodontology, King's College London Dental Institute, Floor 21, Tower Wing Guys Hospital, London SE 1 9RT, UK

## Abstract

*Introduction.* Surgical management of infrabony defects is an invasive procedure, frequently requiring the use of adjunctive material such as grafts or biologics, which is time-consuming and associated with expense and morbidity to the patient. Lasers in periodontal regeneration have been reported in the literature, with each wavelength having potential benefits through different laser-tissue interactions. The purpose of this case series was to assess the efficacy of a new dual-wavelength protocol in the management of infrabony defects.* Materials and Methods.* 32 defects (one in each patient) were treated using ultrasonic debridement, followed by flapless application of Erbium, Chromium:Yttrium, Scandium, Gallium, Garnet (Er,Cr:YSGG) laser (wavelength 2780 nm), and final application of diode laser (wavelength 940 nm). Pocket depths (PD) were measured after 6 months and repeat radiographs taken after one year.* Results.* The mean baseline PD was 8.8 mm (range 6–15 mm) and 6 months later was 2.4 mm (range 2–4 mm), with mean PD reduction being 6.4 ± 1.7 mm (range 3–12 mm). There was a significant gain in relative linear bone height (apical extent of bone), with mean percentage bone fill of 39.7 ± 41.2% and 53% of sites showing at least 40% infill of bone.* Conclusion.* The results compare favourably with traditional surgery and require further validation through randomised clinical controlled trials.

## 1. Introduction

The successful management of periodontal pockets associated with infrabony defects through nonsurgical treatment alone is an unpredictable treatment modality. The optimum outcome of such management would be complete pocket resolution and ideally with new attachment formation. In order to achieve this predictably, several regenerative surgical techniques have been developed, alongside the use of a multitude of both osteoconductive or osteoinductive regenerative materials [1]. New attachment formation is otherwise an inconsistent outcome following nonsurgical treatment alone, although it can happen spontaneously, particularly in cases where defects are deep and narrow.

Lasers in periodontal therapy, both surgically and nonsurgically, are becoming more common, but the evidence base is still highly controversial. Generally, the standard of research has been poor and inconsistent, making a general consensus difficult to reach [2–5]. “Lasers” have been grouped together in the literature rather than looking at each wavelength and its potential tissue interactions, so the benefits of certain wavelengths are often diluted in systematic reviews, and the conclusions have to be taken with caution, accepting the limitations of low numbers of studies and inconsistencies in the protocols and methodology [2, 6].

Several different types of lasers have been proposed as alternatives or adjuncts to conventional surgical periodontal therapy, Nd:YAG [7, 8], Er:YAG [9–11], Er,Cr:YSGG [12], and diode lasers [13], and have shown effective use in regeneration or at least some radiographic evidence of bone fill.

Each has different mechanisms of action, with the Erbium lasers not penetrating so deeply and being less bactericidal, but safe to use on root surfaces and bone, while the diodes and Nd:YAG lasers have greater potential to cause thermal damage to the root surface and bone but are more deeply penetrating with more of a photobiomodulatory effect [11]. Laser energy can be delivered though thin flexible fibres or tips, to sites in the periodontal pocket that conventional instrumentation is less able to reach [14].

While in a specialist practice setting, the author had previously reported on the findings of bone fill using a developed protocol of just the Er,Cr:YSGG laser [15]. Therefore, the purpose of this retrospective case series was to assess the effectiveness of using a dual-wavelength approach with the Er,Cr:YSGG (2780 nm) laser and more deeply penetrating diode laser (940 nm) as an adjunct to conventional nonsurgical therapy, in the resolution of periodontal pockets associated with infrabony defects, which in the majority of cases may have otherwise required flapped regenerative or osseous surgery to treat effectively.

## 2. Materials and Methods

Patients were diagnosed at consultation with either chronic periodontics or aggressive periodontitis based on the 1999 International Classification for Periodontal Diseases and Conditions [16]. Full mouth periapical radiographs were taken at that stage and appropriate risk-related treatment planning was recommended.

**Figure 1:**
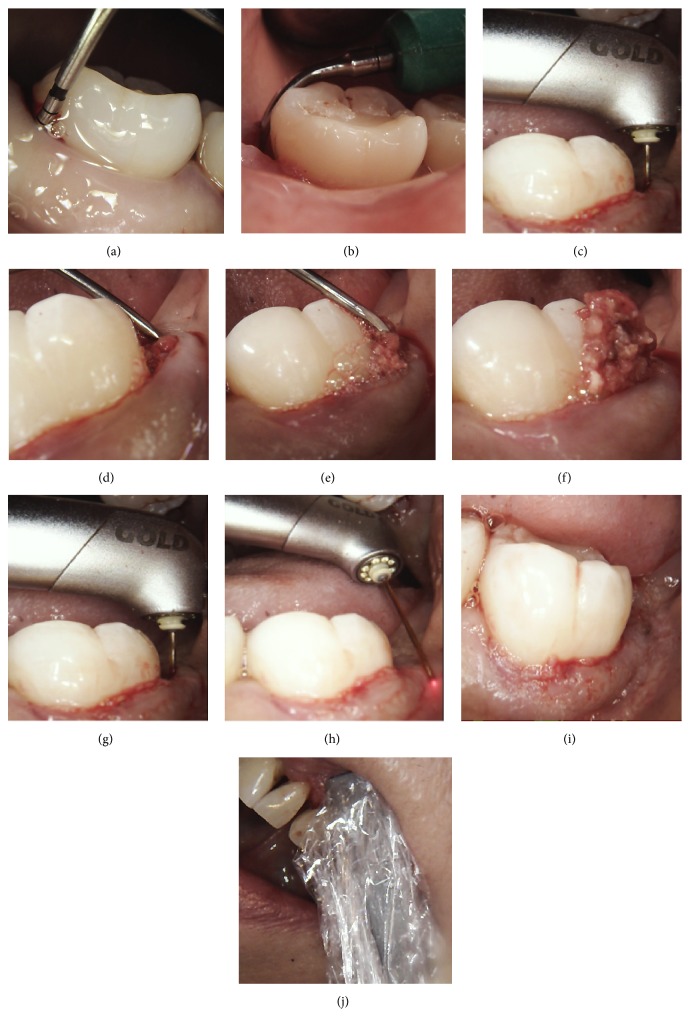
(a) Initial probing depth of 12 mm; (b) subgingival root surface debridement using a Cavitron Slimline tip; (c) first laser application using radial firing tip working from base of pocket upwards; (d, e, f) scraping bone with curette and removal of remaining granulation tissue from the defect; (g) second laser application using radial firing tip to contact the bone, root surfaces, and final debridement of the pocket; (h) external application of laser using radial firing tip to disrupt the external epithelium to a distance equivalent to the pocket depth; (i) immediate post-op after bleeding stopped by compression with damp water-moistened gauze; (j) application of diode laser using a contour handpiece covered by a plastic barrier.

All patients were treated by the same operator, with a combination of conventional root surface instrumentation, followed by Er,Cr:YSGG and then diode laser application. The procedure is shown in Figures 1(a)–1(j). Local anaesthetic was administered at all sites affected by pocketing. The anaesthetic used was 2% lignocaine hydrochloride solution with 1 : 80000 adrenaline and was administered as a combination of buccal and palatal infiltrations and/or inferior dental blocks, to achieve full tooth and soft tissue anaesthesia. Supra- and subgingival debridement were then carried out using ultrasonic scaling tips of varying angles (Dentsply Cavitron, inserts FSI 100, FSI SLI 10 L, and 10 R). Following this, the laser energy was applied.

This was first carried out using a 14 mm, 500-micron radial firing zirconia periodontal tip (Biolase, Irvine California, RFPT5). The settings used were power 1.5 W, frequency 30 Hz, 50% water, 40% air, and H (short pulse 60 *μ*s) mode. The tip was inserted into the base of the pocket and maintained at an angle parallel to the long axis of the root and the epithelial lining as much as possible. Once it touched bone, it was withdrawn slightly and constantly moved vertically (apicocoronal), up and down the pocket, and side to side (either buccolingual or mesiodistal depending on location of the pocket), with slow smooth sweeping motions. This was continued until no further deposits of granulation tissue were seen to be coming out of the pocket. A periodontal curette was then used by scraping it along the bony walls of the defect, to remove any final pieces of granulation tissue. The laser tip was then reinserted and moved slowly and angled this time firstly parallel to the root surface and then towards all the bone surrounding the root, now that the granulation tissue had been removed, the aim being energy contact with all the hard tissues (bone and root surface). The laser tip was run gently along the surrounding bone, as there is a degree of tactile feel with this type of laser and tip. Finally the tip was run outside the pocket, parallel to the tissue, to disrupt the epithelium surrounding the tooth by a distance from the gingival margin equivalent to the depth of the pocket.

If bleeding was excessive following the procedure, pressure with water-moistened damp gauze was applied to the tissues until bleeding stopped.

Next the 940 nm diode laser was used outside the pocket, by holding the contour handpiece at the gingival margin for a period of 20 seconds, both buccally and lingually, at a power setting of 1.4 W. This was equivalent to a dose of 5 J/cm^2^.

Patients were advised to commence brushing as normal the next day and to use appropriate sized interdental brushes. No antibiotics or occlusal adjustment was carried out in any of these cases.

Periodontal reassessment consisting of pocket depth, bleeding on probing, and mobility was carried out at 2 months and 6 months, and periapical radiographs (paralleling technique with holders) were repeated on those sites that were associated with infrabony defects, at a minimum of 12 months after treatment.

All consecutive cases where an infrabony defect had been treated and 12-month follow-up radiographs were available were included in the radiographic and statistical analyses (32 consecutive patients in total). Exclusions were those patients who had conflicting medical histories, such as immune-compromised, or had been treated with adjunctive antibiotics.

**Figure 2:**
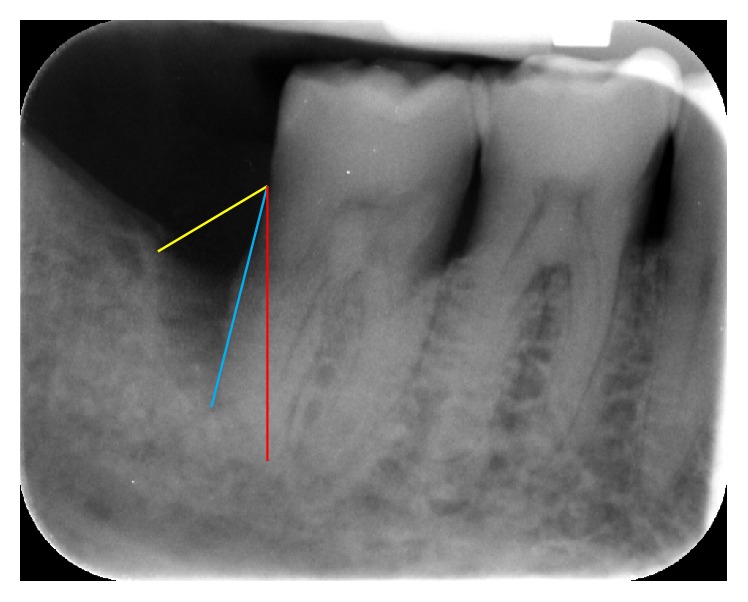
Radiographic measurements to measure changes in relative linear bone height. Root length from CEJ to apex (red line), CEJ to coronal extent of bone height (yellow) on the root surface, and CEJ to most apical extent of bone defect (blue).

Before and after radiographs for each infrabony site were randomly placed side by side on a black viewing background. The radiographs were assessed for relative linear bone height by an independent, blinded examiner (RW) by measuring root length from CEJ to apex, CEJ to coronal extent of bone height (COR) on the root surface, and CEJ to most apical extent of bone on the root surface (API) (as shown in Figure 2). The API and COR measurements were divided by total root length to calculate relative apical and coronal linear bone heights.

## 3. Results

No adverse effects were reported following the treatment, and while no visual analogue scale was used, there was anecdotal reporting of little or no need for analgesics postoperatively in most cases and no postoperative infections requiring antibiotics, and those patients who had previously conventional surgery carried out reported a much more “pleasant” experience.

**Figure 3:**
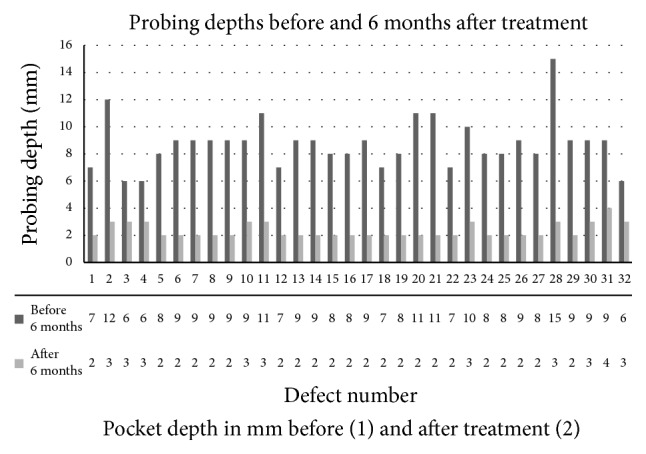
Probing depth in each defect before (series 1) and six months after treatment (series 2). The exact probing depth for each defect is shown in tabulated form below the defect number.

Thirty-two sites associated with infrabony defects, from 32 patients, were included in the analysis. 22 were female and 10 male, with a mean age of 56.7 ± 10.7 years (range 32–79 years). Of those, 3 of the patients were smokers. The mean baseline pocket depth (PD) was 8.8 mm (range 6–15 mm). The mean pocket depth 6 months after treatment was 2.4 mm (range 2–4 mm), and the mean PD reduction was 6.4 ± 1.7 mm (range 3–12 mm). The maximum pocket depth after 6 months was 4 mm. The results for each site are shown in more detail in Figure 3.

**Figure 4:**
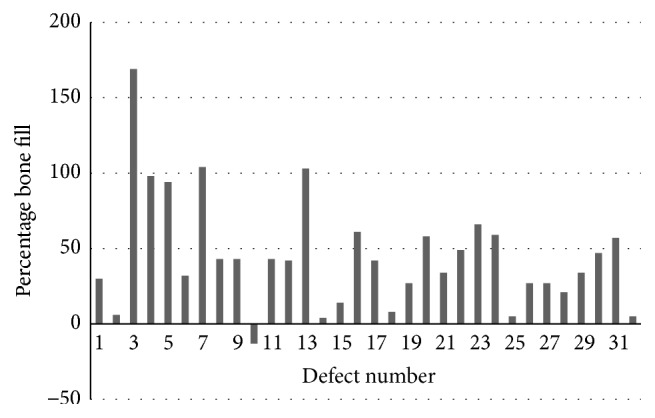
Percentage bone fill in each defect as measured from the radiographical analysis.

**Figure 5:**
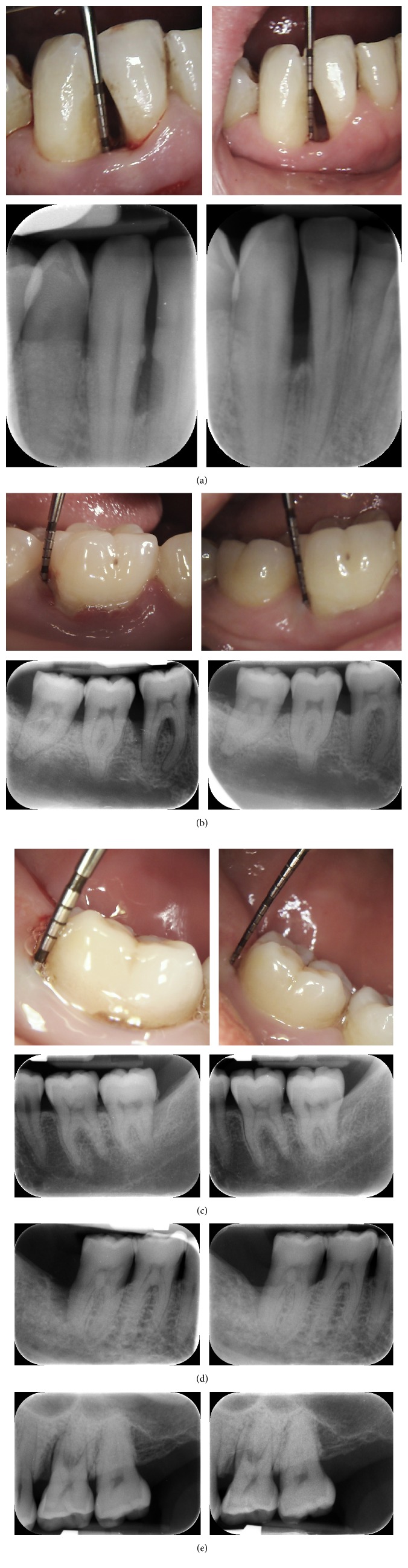
Five cases showing typical results observed. (a) Case 1: 8 mm pocket on the mesial aspect of tooth 42 before treatment, reduced to 3 mm six months later and radiographic bone fill of the defect visible. (b) Case 2: 8 mm defect pre-op associated with an infrabony defect on the distal aspect of 42. The gingivae are inflamed and there is also some recession. Post-operatively, the probing depth is 3 mm, with healthy tissues and minimal recession. The post-op radiograph shows infill of bone distally and in the furcation. (c) Case 3: Pre-op photograph of a 9 mm pocket on the disto-lingual aspect of 37, associated with an infrabony defect as seen on the radiograph. Post-op photograph shows a 2 mm probing depth, with no visible recession having occurred, and the radiograph shows in-fill of bone. (d) Case 4: Pre-op radiograph shows an infrabony defect on the distal aspect of 47 (defect number 28: probing depth was 15 mm). Post-op radiograph shows bone regeneration in the defect (post-op probing depth was 3 mm). (e) Case 5: Defect number 31: pre-op probing depth of 9 mm reduced to 4 mm post-op. There is evidence of radiographic bone fill on the radiograph, but a vertical component of the defect remains, which seems to be the one-wall component of the defect.

There was no significant change in supracrestal bone height. However, there was a significant gain in relative linear bone height (apical extent of bone) in all sites. The mean percentage bone fill was 39.7 ± 41.2%, with 53% of sites showing at least 40% infill of the bony in the infrabony defects. These results are shown in Figure 4. A few of the clinical cases are shown in Figure 5.

## 4. Discussion

The results seen in this case series compare very favourably with other surgical regenerative therapies [17, 18] and would suggest that this minimally invasive technique with adjunctive use of two laser wavelengths is an effective treatment modality for the management of infrabony defects. Each laser wavelength has a different tissue interaction, and so the potential benefits of using two laser wavelengths together may be greater than one alone.

The radial firing tips are ideal for the use in periodontal pockets due to their ability to fire laser energy laterally onto the root surface and also the pocket lining. In doing so, they are effective in the removal of biofilm from the root surface without causing thermal or mechanical damage [19, 20] and, at the same time, are able to ablate soft tissue and have a bactericidal effect.

The Er,Cr:YSGG wavelength (2780 nm) is close to the peak of absorption coefficient of water; therefore the absorption of the energy occurs rapidly, resulting in evaporation of water, microexplosive ablation, and reduced heat accumulation. The high coefficient of absorption of the light frequency by lipopolysaccharides gives it its bactericidal effect and has been found to have a significant bactericidal effect on both* P. gingivalis* and* A. actinomycetemcomitans*, both periopathogenic pathogens [14, 21]. It can be used safely in periodontal pocket therapy and has been claimed in the literature to provide a more comfortable patient experience, with fewer postoperative complications, and faster wound healing [22, 23]. Some studies have gone so far as to conclude that the use of this laser wavelength may be a suitable alternative to conventional open flap debridement in the management of deep pockets and patients with advanced chronic periodontitis [22]. However, the literature is still in its infancy and the majority of systematic reviews that have considered this wavelength have concluded that there are not enough studies or consistency among them to conclude that laser treatment is in any way superior to conventional periodontal therapy [2, 6].

Besides having a bactericidal effect, removal of biofilm, smear layer, endotoxin, and calculus [24–28], and ability to remove granulation tissue, the Er,Cr:YSGG also modifies the root surface in such a way as to be more favourable for the attachment of fibroblasts and blood components, compared to scaled (ultrasonic or hand instrumented) root surfaces [24, 29, 30]. The removal of the outer epithelium is perhaps acting similarly to a periodontal membrane, delaying the downgrowth of epithelial cells and allowing more time for a connective tissue attachment to form, which takes five times longer [31]. Indeed, some animal studies using the CO_2_ laser in the removal of outer epithelium demonstrated histological evidence of periodontal regeneration [32, 33]. The action of the laser on the bone, with resultant bleeding, also promotes the release of stimulatory cytokines and growth factors [9, 11, 12], which may all have played a role in the resultant bone growth seen in these cases.

In recent periodontal regeneration studies there has been great emphasis on the importance of wound stability and indeed the use of minimally invasive surgical techniques has been shown to result in equivalent regenerative outcomes even without the application of grafts and other regenerative materials [34–36]. In the use of the laser described here, debridement and granulation tissue removal are carried out flaplessly which may result in equivalence to minimally invasive surgical techniques assuming adequate wound debridement. Yukna et al. 2007 and Nevins et al. 2012 [7, 8] both demonstrated histological evidence of regeneration using Nd:YAG lasers, with an important part of the protocol being the achievement of a blood clot and wound stability.

Diode lasers are known to have a bactericidal effect, particularly on pigmented periodontal pathogens [37, 38]. However, the timing, application, and dose used in these cases allowed more for a potential biostimulatory effect due to its deeper penetration than Erbium lasers, with very little, if any, rise in temperature [39, 40]. Possible cellular mechanisms encouraged by the use of the diode laser in this way and with this dosing include stimulation of periodontal ligament stem cells; stimulation of fibroblasts; and osteoblast cell differentiation [41–44]. Cytokine stimulation and regulation aid in regeneration and reduce inflammation. Sadighi 2012 [45] showed that low level laser therapy resulted in additional benefits to bone grafting alone in 2- and 3-wall infrabony defects, with faster regeneration, greater pocket depth reduction, and less bleeding on probing. Doğan et al. 2014 [13] also found greater clinical benefits when combining GTR with low level laser therapy, at similar doses to those used in this study.

Therefore, the combination of the two laser wavelengths provides different laser-tissue interactions, compared to using just one wavelength alone, which may have been of benefit in these cases. The Erbium laser is primarily of surgical benefit, on soft tissue, root surface, and bone, along with a bactericidal and root surface modifying effect that can play a role in wound healing; and the diode potentially has a primarily biostimulatory effect, contributing to cytokine regulation and playing a role in the wound healing process. The minimally invasive technique saves time and expense for the practitioner and saves expense for the patient, compared to flapped surgical treatment, and avoided the use of expensive grafting materials. Patient centred outcomes are considered to be factors of increasing importance and there is a significant need for more of this to be reported in the literature [46, 47]. The protocol produced predictable results as demonstrated by the results seen in a large number of cases treated the same way and warrants incorporation into double-blinded randomised clinical controlled trials for validation and further investigation.
